# Supplementary material for: Beta-Glucan modulates monocyte plasticity and differentiation capacity to mitigate DSS-induced colitis
Source: eLife. 2026 Jun 1;14:RP107339. doi: 10.7554/eLife.107339 (PMC13225843; doi:10.7554/eLife.107339)
Supplement: Supplementary file 1. [file elife-107339-supp1.docx]

| Antibody | Clone No. | | Cat. No. | Company |
| --- | --- | --- | --- | --- |
| Biotin anti-mouse CD4 antibody | | GK1.5 | 100404 | Biolegend |
| Biotin anti-mouse CD8 antibody | | 53-6.7 | 100704 | Biolegend |
| Biotin anti-mouse Ly6G antibody | | 1A8 | 127604 | Biolegend |
| TruStain FcX^TM^ (anti-mouse CD16/32) antibody | | 93 | 101320 | Biolegend |
| Anti-mouse CD45 antibody, PerCP-Cyanine5.5 | | 30-F11 | 45-0451-82 | eBioscience |
| Anti-mouse CD45 antibody, APC | | 30-F11 | 17-0451-82 | eBioscience |
| Anti-mouse CD45 antibody, Brilliant Violet 711^TM^ | | 30-F11 | 103147 | Biolegend |
| Anti-mouse CD45.1 antibody, PE | | A20 | 12-0453-82 | Invitrogen |
| Anti-mouse CD45.1 antibody, APC-eFluor 780 | | A20 | 47-0453-82 | Invitrogen |
| Anti-mouse CD45.1 antibody, FITC | | A20 | 11-0453-82 | Invitrogen |
| Anti-mouse CD45.2 antibody, PE-Cyanine7 | | 104 | 25-0454-82 | Invitrogen |
| Anti-mouse CD45.2 antibody, FITC | | 104 | 11-0454-81 | Invitrogen |
| Anti-mouse CD4 antibody, eFluor450 | | GK1.5 | 48-0041-82 | eBioscience |
| Anti-mouse CD4 antibody, PE-Cyanine7 | | RM4-5 | 25-0042-82 | eBioscience |
| Anti-mouse CD8a antibody, Brilliant Violet 605™ | | 53-6.7 | 100744 | Biolegend |
| Anti-mouse CD19 antibody, PE | | 1D3/CD19 | 152408 | Biolegend |
| Anti-mouse CD192 Antibody, PE | | SA203G11 | 150610 | BioLegend |

| Antibody | Clone No. | | Cat. No. | Company |
| --- | --- | --- | --- | --- |
| Anti-mouse CD11b antibody, eFluor 450 | | M1/70 | 48-0112-82 | eBioscience |
| Anti-mouse CD11b antibody, PE-Cyanine7 | | M1/70 | 25-0112-82 | Invitrogen |
| Anti-mouse Ly-6G antibody, PE | | 1A8 | 12-9668-82 | eBioscience |
| Anti-mouse Ly-6G antibody, Brilliant Violet 421™ | | 1A8 | 127628 | Biolegend |
| Anti-mouse CX3CR1 antibody, Pacific Blue™ | | SA011F11 | 149037 | Biolegend |
| Anti-mouse CX3CR1 antibody, Alexa Fluor^TM^ 647 | | Z8-50 | 567805 | BD |
| Anti-mouse Ly-6C antibody, Brilliant Violet 605™ | | HK1.4 | 128036 | Biolegend |
| Anti-mouse Ly-6C antibody, PE/Dazzle™ 594 | | HK1.4 | 128044 | Biolegend |
| Anti-mouse Ly-6C antibody, eFluor 450 | | HK1.4 | 48-5932-82 | Invitrogen |
| Anti-mouse MHC Class II (I-A/I-E) antibody, PE/Cyanine5 | | M5/114.15.2 | 15-5321-82 | eBioscience |
